# Micropollutants in treated wastewater

**DOI:** 10.1007/s13280-019-01219-5

**Published:** 2019-07-10

**Authors:** Justyna Rogowska, Monika Cieszynska-Semenowicz, Wojciech Ratajczyk, Lidia Wolska

**Affiliations:** Department of Environmental Toxicology, Faculty of Health Sciences, Medical University of Gdansk, Debowa Str. 23A, 80-204 Gdansk, Poland

**Keywords:** Aquatic environment, Ecotoxicity, Microorganic pollutants, Wastewater, Wastewater treatment plants (WWTPs)

## Abstract

Compounds such as pharmaceuticals, or personal care products are only partially removed in wastewater treatment processes. Large number of these compounds and their degradation products is out of any control. A small number of compounds are covered by legal regulations. Among the compounds non-regulated by law, the target compounds, as well as non-target compounds can be distinguished. In the scientific literature, number of reports on various target compounds’ determination is increasingly growing. This paper provides an up-to-date review on micropollutants present in treated wastewater and their concentrations found in literature in the years 2015–2019. Because the obtained results of chemical analyses do not adequately reflect the risks to ecosystems and consequently humans, the results of chemical analyses have been supplemented by a review of ecotoxicological studies. In addition, legal issues linked to contamination of treated wastewater and research related to identification of non-target compounds in treated effluents have been discussed.

## Introduction

The global socioeconomic development generates a stream of substances (some of them are new), which almost immediately occur in the environment. It has been estimated, that the chemical industry currently produces more than 70 000 different chemical products, with an estimated worldwide sales value of $5000 billion (Asthana 2014). Many of these substances released into the aquatic environment pose a serious risk for the environment and for human health.

**Fig. 1:**
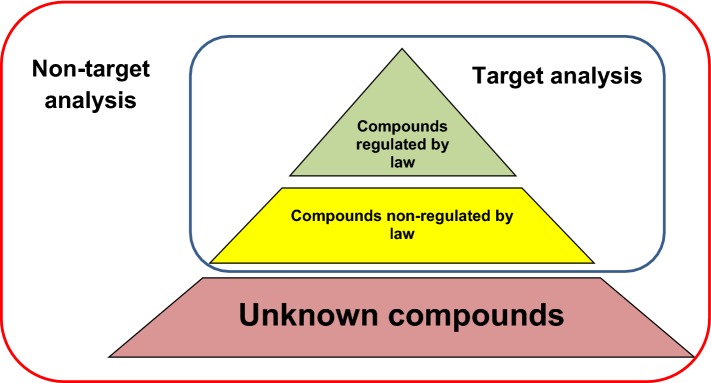
Types of compounds found in the aquatic environment

In the last decade, the political awareness of water quality issues has grown substantially in the European Union (EU), as wastewater treatment plants (WWTPs) have been identified as a major point source pollution (Corominas et al. 2013). Conventional WWTPs are incapable of eliminating many compounds found in wastewater. In last decades, much attention has been paid to analytics compounds such as endocrine-disrupting chemicals (EDCs) or pharmaceuticals. Treated wastewater released from WWTPs can be discharged to the receiving bodies such as surface waters (e.g., rivers, lakes) or, preferably from the end of the last century in some regions, sea waters. As a consequence, many compounds found in wastewater effluents and/or their metabolites and transformation products are detected in surface waters and to great concern of scientists, end up in marine environment. The properties of these substances and their impact on the environment and human health are often unknown. Knowledge about the long-term effects of exposure to a mixture of pollutants present in the environment at low concentration levels is still limited (HELCOM 2003). It should be noted that only substances that are commonly found in the environment at a significant concentration levels and at the same time posing a threat to the environment and/or human health are covered by legal norms (compounds regulated by law). For example, according to Art. 16 of the Water Framework Directive is the list of priority substances that pose a threat to the aquatic environment. This risk is assessed according to a procedure based on scientific principles. To include the substance in the list of priority substances in the field of water policy, a reliable scientific evidence must be provided ‘*regarding the intrinsic hazard of the substance concerned, and in particular its aquatic ecotoxicity and human toxicity* via *aquatic exposure routes, and evidence from monitoring of widespread environmental contamination, and other proven factors which may indicate the possibility of widespread environmental contamination, such as production or use volume of the substance concerned, and use patterns*’ (WFD 2000). Therefore, only a small number of compounds is covered by the legal regulations (Fig. 1). These compounds are systematically monitored in the environment, e.g., polychlorinated biphenyls (PCBs). But large number of compounds and their degradation products fall outside of any control. Among the non-regulated by law compounds, compounds which can be expected in the wastewater due to their considerable emission into the environment can be distinguished. Currently, these are mostly pharmaceuticals. The second and most numerous group constitutes unknown compounds. The number of potential contaminants is essentially endless. For example, over 10 000 prescription drugs and about 300 over-the-counter drugs are currently in use and produced in USA and may be released to the environment during processing or use (Dong et al. 2013). Furthermore, degradation and transformation products of certain substances in the environment can have unknown structure and properties. The newly formed, emerging, products may pose a greater threat to the environment (and organisms living in it) than the parent compounds (Garnaga 2012). It should also be noted that in an aquatic environment, substances are present in the mixtures and still there is a lack of comprehensive knowledge about the effects of chemicals, their combinations/mixtures on the environment and human health.

The aim of our study was to review literature on the presence of contaminants in treated sewage and their highest concentrations. We point out that the research articles on the pollutants present in wastewater are primarily based on target analysis. On the other hand, there are very few research papers covering problems related to identification of non-target compounds in treated effluents. What is more, we have indicated threats to aquatic ecosystems as a consequence of the presence of toxic compounds and endocrine active compounds in treated effluents on the basis of ecotoxicological studies. The information has been supplemented with legal issues linked to contamination of treated wastewater and research (and problems) related to identification of non-target compounds in wastewater effluents.

## Methods

In this review, we have focused on recent studies published from years 2015–2019. We searched Scopus database, which provides access to STM journal articles and the references included in those articles. We entered a combination of terms/keywords such as ‘micropollutants,’ ‘concentration,’ ‘effluents,’ and ‘wastewater’ into search box and sorted results by relevance. In literature, we were looking for the highest measured and reported concentrations of compounds in effluents from conventional wastewater treatment plants worldwide, collecting a mixture of domestic and industrial wastewater. In our review, we do not include concentrations of compounds found in effluents coming from industry alone (e.g., pharmacy, slaughter house, pulp and paper, textiles, hospital effluents), wastewater influents, potable water, surface waters. Due to the fact that for each year we found over 600 references and the resources are virtually infinite, the present review is a selection of just some of the major studies. Our selection of micropollutants thus must focus on chemicals that in our opinion pose the greatest threat to environment is due to the following: high measured concentrations, significant ecotoxicity, frequency of detection, and unsatisfactory removal efficiency. For the micropollutants with the highest concentrations detected, we compiled ecotoxicity data based on laboratory results.

The occurrence of micropollutants in wastewaters was revised by many authors: Das et al. (2017), Jiang et al. (2013), Luo et al. (2014), Petrie et al. (2015), and Ratola et al. (2012). These reviews mainly indicate compounds detected in treated wastewaters, and efficacy and methods of wastewater treatment. Therefore, in our review we decided to fill in the knowledge gap and added ecotoxicity data for the micropollutants with the highest measured concentrations mentioned in literature.

## Micropollutants in treated wastewater

### Legal aspects of contaminants in treated wastewater

In Europe, the state of the aquatic environment is controlled by legislation outlined by the European Commission. Directive 2000/60/EC setting out the framework for community action in the field of water policy has reformed the water quality policy of the community, and is the first attempt to move towards ecosystem-based management that should ensure the good ecological status (WFD 2000; Corominas et al. 2013). Its aim is the prevention of water pollution within the European Union through such steps as identifying the pollutants which pose the greatest risks to or via the water environment. The priority under this directive is to identify and eliminate the sources of harmful emissions. A supplementary of WFD is Directive 2008/105/EC of the European Parliament and of the Council of 16 December 2008 on environmental quality standards in the field of water policy and Directive 2013/39/EU of the European Parliament and of the Council of 12 August 2013 amending Directives 2000/60/EC and 2008/105/EC as regards priority substances in the field of water policy which includes the list of 45 priority substances. Enactment in 2008 Directive 2008/56/EC of the European Parliament and of the Council of 17 June 2008 establishing a framework for community action in the field of marine environmental policy called the Marine Strategy Framework Directive (MSFD), widens the scope of the European Union (EU) legal framework to cover the marine environment for a new EU-integrated ecosystem policy for the protection of the water environment.

Requirements for the quality of wastewater discharged from the plant are included in Council Directive 91/271/EEC of 21 May 1991 concerning urban wastewater treatment. Directive determines the inter alia requirements for discharges from urban wastewater treatment plants, including emission limit values for these. Treated wastewater discharged from the WWTPs are characterized only by chemical and biochemical oxygen demand and total suspended solids. In the case that treated effluents from WWTPs are discharged to sensitive areas which are prone to eutrophication, provisions of the Directive require also the determination of total nitrogen and total phosphorus. Specifically, WWTPs effluents are controlled by a ‘combined’ approach of emission limit values, load reduction, and environmental quality standards, along with the restriction or phasing out of particularly priority and dangerous priority substances under these Directives (Corominas et al. 2013).

Analyzing the provisions of Directives it should be noted that:

1. The European Commission carries out a regular review of a list of priority substances in the field of water policy. The European Commission also prepares a list of observational material. Substances to be placed on the watch list are selected from among those for which the available information suggests that they may represent the significant risk to the aquatic environment or through, and for which monitoring data are insufficient (WFD 2000). However, what has already been mentioned, for the relatively small number of organic pollutants in the environment changes are fully understood, and the majority of these impurities cannot be identified (HELCOM 2003).
2. Treated wastewater discharged from the WWTPs are characterized by total nitrogen/phosphorus, total suspended solids, and chemical/biochemical oxygen demand.
3. Legal provisions do not take into account the interactions between pollutants (even those included in the list of priority substances) such as synergism, additivity, or antagonism.

### Contaminants in treated wastewater

Conventional secondary processes (activated sludge and trickling filters) represent the most extensively used method of wastewater purification. However, these processes fail to remove large number of chemical compounds. For example, some pharmaceuticals such as paracetamol or ibuprofen are efficiently removed through conventional treatment methods (> 99% and 72–100%, respectively) (Ratola et al. 2012; Luo et al. 2014), while others, such as sulfamethazine or carbamazepine are being removed from wastewater less effectively (13% and 7–23%, respectively) (Ratola et al. 2012). As a result, compounds belonging to groups of pharmaceuticals, personal care products, surfactants, biocides, or flame retardants may be released to surface waters (rivers, lakes, or coastal waters) (Petrie et al. 2015). Many pharmaceuticals may undergo various transformations in the environment, animal, or human body. Pharmaceuticals can be completely or partially metabolized in the organisms, what may lead to the unchanged parent drugs and the produced metabolites excretion via urine and/or feces (Ribeiro et al. 2016). Particular interest is also aroused by transformation products that can be formed during water disinfection and wastewater treatment, as well as due to various processes occurring in natural waters such as biodegradation, photodegradation, hydrolysis (Nikolaou 2013; Deeb et al. 2017). What is more, compounds found in wastewater can degrade and/or react with other compounds in the environment re-emitting products of higher toxicity than the original compounds. The determination of the toxic effects of pharmaceuticals, their transformation products and mixtures in the environment, is a subject requiring urgent attention, and a great challenge for scientists. Moreover, they are present in trace concentrations (Nikolaou 2013). Number of papers related to multiresidue analytical methodologies has increased over recent years; however, most of them are focused on target analysis methods (Kotowska et al. 2014; Gurke et al. 2015; García-Galán et al. 2016; Knopp et al. 2016; Roberts et al. 2016; Madikizela and Chimuka 2017; Petrie et al. 2017; Wang et al. 2018).

Potential risks of adverse effects caused by effluents from WWTPs to aquatic environments are influenced by volume of effluent, concentration of compounds in wastewater, the water flow rate of the receiving river, weather conditions, and probably other factors that affect dissipation through dilution and/or degradation. The compounds detected in effluents from sewage-treatment plants at concentrations above 1 µg/L and published in the period of 2015–2019 are listed in Table 1. For our list, we established a limit of concentration recognized as environmentally relevant in prioritization of contaminants in wastewaters (Blum et al. 2017; Gros et al. 2017).

**Table 1:** The maximum concentrations of most often determined compounds in effluents from WWTPs, arranged in order of decreasing concentration

Group of compounds	Identified compounds	Highest concentration determined (µg/L)	References
Antidepressant agents	Citalopram	840	Cunha et al. (2019)
Nanoparticles	Nanoscale fragments containing 70-85% of carbon, low amounts of oxygen and heavy metals	550 ± 130	Hu et al. (2018)
Antiepileptics	Gabapentin	79.86	Oliveira et al. (2015)
Analgesics/anti-inflammatories	Tramadol	59.05	Petrie et al. (2015)
Antiretroviral agents	Lamivudine	55.76 ± 5.48	Ngumba (2018)
Antiretroviral agents	Zidovudine	37.14 ± 2.56	Ngumba (2018)
Antiretroviral agents	Efavirenz	34 ± 2.8	Abafe et al. (2018)
H2-receptor antagonists	Valsartan	28.22	Gurke et al. (2015)
Metabolites	*N*-acetyl-4-aminoantipyrine	25.03	Evgenidou et al. (2015)
Industrial chemicals	4-Methyl-1H-benzotriazole	24.30	Deeb et al. (2017)
Analgesics/anti-inflammatories	Diclofenac	23.50	Madikizela and Chimuka. (2017)
Artificial sweetener	Acesulfame	22.50	Das et al. (2017)
Industrial chemical	1H-benzotriazole	22.10	Deeb et al. (2017)
Artificial sweetener	Sucralose	18.80	Tolouei et al. (2019)
Angiotensin receptor antagonist	Irbesartan	17.90	Kårelid et al. (2017)
Contrast media	Iopromide	17.90	Qi et al. (2015)
Antiretroviral agents	Darunavir	17 ± 0.55	Abafe et al. (2018)
Contrast media	Iopamidol	16.29	Völker et al. (2017)
Anti-anxiety agents	Bromazepam	15.54	Cunha et al. (2019)
Analgesics/anti-inflammatories	Naproxen	14.40	Madikizela and Chimuka. (2017)
Analgesics/anti-inflammatories	Acetaminophen	11.73	Petrie et al. (2015)
Contrast media	Diatrizoate	11.73	Völker et al. (2017)
Stimulants	Caffeine	11.45	Gros et al. (2017)
Metabolites	Metronidazole-OH	11.34	Evgenidou et al. (2015)
Contrast media	Iomeprol	11.25	Völker et al. (2017)
Antidiabetic drugs	Metformin	10.35	Das et al. (2017)
Diuretics	Furosemide	9.96	Papageorgiou et al. (2016)
Analgesics/anti-inflammatories	Nimesulide	9.73	Papageorgiou et al. (2016)
Metabolites	4-Aminoantipyrine	9.29	Evgenidou et al. (2015)
Metabolites	4-Methylaminoantipyrine	9.25	Evgenidou et al. (2015)
Analgesics/anti-inflammatories	Ibuprofen	9.20	Gros et al. (2017)
Metabolites	Erythromycin-H_2_O	7.84	Evgenidou et al. (2015)
Anti-anxiety agents	Oxazepam	7.43	Cunha et al. (2019)
Contrast media	Diatrizoic acid	7.03	Ribbers et al. (2019)
Metabolites	4′-Hydroxy diclofenac	7.02	García-Galán et al. (2016)
Beta-blockers	Metoprolol	5.76	Gurke et al. (2015)
Metabolites	Erythro/threo-hydrobupropion	5.70	Evgenidou et al. (2015)
Metabolites	o-desmethylvenlafaxine	5.50	Evgenidou et al. (2015)
Antidepressant agents	Venlafaxine	5.50	Roberts et al. (2016)
Analgesics/anti-inflammatories	Codeine	5.27	Petrie et al. (2015)
Analgesics/anti-inflammatories	Ketoprofen	5.25	Oliveira et al. (2015)
Antibiotics	Cephalexin	5.07	Deeb et al. (2017)
Flame retardants	Tri-(2-chloroisopropyl)phosphate	4.90	Gros et al. (2017)
Analgesics/anticonvulsant	Carbamazepine	4.61	Deeb et al. (2017)
Flame retardants	Tris-(2-butoxyethyl)phosphate	4.60	Gros et al. (2017)
Sunscreen Agent	4-Benzophenone	4.31	Petrie et al. (2015)
Preservative and anti-infective agent	Triclosan	4.26	Deeb et al. (2017)
Industrial chemicals	2,4,7,9-Tetramethyl-5-decyne-4,7-diol	4.20	Blum et al. (2017)
Antiepileptics	Lamotrigine	4.12	Oliveira et al. (2015)
Diuretics	Theobromine	4.01	Oliveira et al. (2015)
Antiretroviral agents	Lopinavir	3.8 ± 0.35	Abafe et al. (2018)
Metabolites	10,11-Dihydro-trans-10,11-dihydroxy-carbamazepine	3.60	Evgenidou et al. (2015)
Metabolites	Carbamazepine-10,11-epoxide	3.58	Evgenidou et al. (2015)
Antiretroviral agents	Raltegravir	3.5 ± 1.3	Abafe et al. (2018)
Diuretics	Hydrochlorothiazide	3.42	Oliveira et al. (2015)
Transformation product (oxidation)	Carboxy-Acyclovir	3.40	Knopp et al. (2016)
Beta-blockers	Sotalol	3.33	Roberts et al. (2016)
Antibiotics	Sulfamethoxazole	3.25	Oliveira et al. (2015)
Bronchodilator	Theophylline	3.17	Petrie et al. (2015)
Lipid regulator	Bezafibrate	3.12	Gros et al. (2017)
Metabolites	Cotinine	3.10	Evgenidou et al.,^2015^)
Beta-blockers	Atenolol	2.87	Deeb et al. (2017)
Angiotensin receptor antagonist	Telmisartan	2.75	Gurke et al. (2015)
H2-receptor antagonists	Cimetidine	2.61	Petrie et al. (2015)
Metabolites	Metoprolol acid	2.51	Evgenidou et al. (2015)
Antibiotics	Trimethoprim	2.40	Deeb et al. (2017)
Flame retardant	Tris(2-butoxyethyl)phosphate	2.40	Blum et al. (2017)
Antibiotics	Penicillin G	2.22	Deeb et al. (2017)
Industrial chemicals	Tolyltriazole	2.20	Knopp et al. (2016)
Antibiotics	Levofloxacin	2.19	Deeb et al. (2017)
Analgesics	Salicylic acid	2.18	Evgenidou et al. (2015)
Angiotensin receptor antagonist	Candesartan	1.99	Gurke et al. (2015)
Antiretroviral agents	Nevirapine	1.9 ± 0.68	Abafe et al. (2018)
Metabolites	10-Hydroxy-10,11-dihydrocarbamazepine	1.90	Evgenidou et al. (2015)
Psychoanaleptics	Desmethylvenlafaxine	1.87	Oliveira et al. (2015)
Metabolites	Guanylurea	1.86	Evgenidou et al. (2015)
Antibiotics	Clarithromycin	1.79	Deeb et al. (2017)
Lipid-regulators	Simvastatin	1.74	Papageorgiou et al. (2016)
Analgesics/anti-inflammatories	Aminopyrine	1.68	Deeb et al. (2017)
Anti-allergic agents	Fexofenadine	1.61	Archer et al. (2017)
Metabolites	Benzoylecgonine	1.60	Petrie et al. (2015)
Metabolites	4′-Hydroxy aceclofenac	1.60	Evgenidou et al. (2015)
Flame retardant	Tris(1-chloro-2-propyl)phosphate	1.60	Blum et al. (2017)
Antiretroviral agents	Ritonavir	1.50 ± 0.053	Abafe et al. (2018)
Antibiotics	Norfloxacin	1.50	Deeb et al. (2017)
Phytosterols	Beta-sitosterol	1.50	Wang et al. (2018)
Metabolites	*O*-Desmethyltramadol	1.47	Archer et al. (2017)
Industrial chemicals	Methylindole	1.42	Deeb et al. (2017)
Beta-blockers	Labetalol	1.40	Oliveira et al. (2015)
Solvents	2-Butoxyethanol	1.40	Wang et al. (2018)
Antibiotics	Erythromycin	1.39	Petrie et al. (2015)
H2-receptor antagonists	Ranitidine	1.38	Dasenaki and Thomaidis (2015)
Hormones	Progesterone	1.34	Deeb et al. (2017)
Metabolites	Carboxy-ibuprofen	1.27	Evgenidou et al. (2015)
Antihistamines	Cetirizine	1.24	Papageorgiou et al. (2016)
Antiepileptics	Pregabalin	1.24	Gurke et al. (2015)
Flame retardant/plasticizer	Tris(2-chloroethyl)phosphate	1.16	Wang et al. (2018)
Antibiotics	Ciprofloxacin	1.08	Deeb et al. (2017)
Angiotensin receptor antagonist	Eprosartan	1.04	Gurke et al. (2015)
Analgesics/anti-inflammatories	Lidocaine	1.00	Oliveira et al. (2015)

The compounds of the highest concentrations in treated effluents are antidepressant citalopram, antiepileptic gabapentin, anti-inflammatory tramadol and diclofenac, and antiretroviral drugs such as lamivudine, zidovudine, efavirenz, and darunavir. It is worth mentioning that one metabolite, *N*-acetyl-4-aminoantipyrine, a metabolite of dipyrone was found in effluent at concentration as high as 25.03 µg/L. Among other compounds with high concentrations also artificial sweeteners acesulfame and sucralose are detected at concentrations of 22.50 µg/L and 18.80 µg/L, respectively (Das et al. 2017; Tolouei et al. 2019). More than 95% of ingested sucralose is excreted in urine, < 2% is degraded at wastewater treatment plants, and the rest is exported unaltered with effluent (Amy-Sagers et al. 2017). Also X-ray contrast media such as iopamidol, iopromide, iomeprol are not effectively eliminated from the wastewater (Santos et al. 2010).

Other micropollutants, which are detected in wastewater effluents, are nanoparticles (NP). Trace amounts of nanoparticles exist naturally in environment; however, recently, their incorporation in technology, medicine, and in many domestic consumer products, contribute to their presence in wastewaters and their inevitable release to aquatic ecosystems. The definition of NP stating that it is any material with at least one dimension in the range of 1–100 nm is very broad. Therefore, many substances such as metals (Ag, Zn, Ni, Fe, Cu); metal oxides (TiO_2_, Fe_3_O_4_, SiO_2_, CeO_2_, and Al_2_O_3_); non-metals (silica and quantum dots); forms of carbon (nanotubes, fullerene, graphene) exist in nanoscale (Madeła et al. 2016). Therefore, even though concentration of nanoscale fragments noted in effluent was significantly high (550 ± 130 µg/L), the effluent content of specific nanoparticles is lower: 5.5 µg/L for Ag-NP, 19.1 µg/L for fullerene C_60_, 1.65 µg/L for fullerene C_70_, and 31.9021 µg/L for *N*-methylfulleropyrrolidine C_60_ (Farré et al. 2010; Yang et al. 2012).

Industrial chemicals, such as 1H-benzotriazole and 4-methyl-1H-benzotriazole, used in a wide range of commercial and industrial applications such as corrosion inhibitors, dishwasher detergents, and antifreezes are also among high-concentration micropollutants of 22.1 µg/L and 24.3 µg/L content, respectively (Deeb et al. 2017).

Out of all the groups of compounds identified and determined in treated effluents, much attention is being paid to the presence of most commonly prescribed antibiotics (ciprofloxacin, doxycycline, norfloxacin, trimethoprim, and sulfamethoxazole) and analgesics/anti-inflammatory pharmaceuticals such as ibuprofen, naproxen, or diclofenac (Deblonde et al. 2011). In addition, in treated wastewater, often triclosan, an ingredient in personal hygiene and household products such as soaps, toothpaste, mouthwash, deodorants, detergents, and disinfecting lotions is detected (Kotowska et al. 2014).

### Non-target analysis

Target analysis is focused on identification and quantification of certain compounds. However, quantification of target chemicals in the treated wastewater is insufficient for risk assessment, due to introduction of wastewater into the environment. The wastewater may contain many unknown substances. Screening of unknown organic compounds present in the treated wastewater allows for the identification of particularly hazardous compounds and can be useful for maintaining the suitable purity of surface water (Kotowska et al. 2012). Most scientific research focuses on the determination of specific compounds. There are only few reports in literature on the identification of non-target contaminants in the treated sewage (Gómez et al. 2009; Kotowska et al. 2012; Hug et al. 2014; Dsikowitzky et al. 2015; Hrubik et al. 2016; Blum et al. 2017; Gros et al. 2017). This is due to the fact that the analysis of these kinds of contaminants is complicated, time consuming, and represents a real challenge for environmental analysts. Non-target analysis allows for identification of both known and unknown chemicals. The analytical methods for the detection and quantification of non-target contaminants (in group of organic compounds) are generally based on separation methods, particularly gas chromatography (GC) or/and liquid chromatography (LC) coupled with a potential instrument for identification such as mass spectrometry (MS) (Gómez et al. 2009). The choice of the applied method is associated with physicochemical properties of the target analytes. Liquid chromatography–high-resolution mass spectrometry (LC–HRMS) offers the possibility to detect hundreds of polar and non-polar compounds without pre-selection of analytes (Hug et al. 2014).

In general for the analysis of pharmaceuticals in wastewater, it is appropriate to use GC; however, degradation products of some of those compounds may be thermolabile and decompose during GC analysis as it is in the case of carbamazepine and its degradation product iminostilbene (Gómez et al. 2009). The compound’s spectrum that is detectable with the use of GC–MS method is restricted to volatile, low-molecular weight non-polar to semi-polar organic substances (Dsikowitzky et al. 2015). Additionally, the use of GC × GC allowed better separation of the analytes from interferences in complex samples without extensive sample preparation (Blum et al. 2017). The disadvantage of GC–MS application is that it requires a time-consuming derivatization step, during which there are risks of analyte losses (Nikolaou 2013). For identification of non-target compounds present in the treated wastewater a mass spectrometry scanning mode full-scan mode can be applied. Another method used, often for quantitative determination, is selected ion monitoring (SIM). The main advantage of the full-scan mode over the SIM mode is the possibility of simultaneous identification of various eluted compounds that could be of interest (Gómez et al. 2009). A major disadvantage is that, generally, the full-scan method is less sensitive than SIM mode, although new generation equipment yields sufficient sensitivity (Gómez et al. 2009). Considerable problems are also attributed to the analysis of the obtained chromatograms, as in non-target screening, in which often no initial information on the analytes is available, automated peak detection and spectra deconvolution algorithms are applied, which typically reveal several thousands of peaks in an individual wastewater sample (Hug et al. 2014).

### Environmental effects

Environmental risks posed by substances depend on their physical and chemical speciation and affinity for solid matter and water, which can have a significant impact on their bioavailability. Furthermore, the risk for living organisms is also dependent on the mobility of substances and their ability to be transferred up in the food chain. In the tissues of marine organisms contaminants can be accumulated or ingested from water or suspended matter. The result is that the pollutants concentration in the tissues of living organisms may be present at levels comparable to the concentrations in the environment or even higher (bioaccumulation). The wide variation in environmental conditions in different water areas can also affect bioavailability. Among these conditions: salinity, temperature, pH changes, or turbidity can be distinguished. In addition to the physicochemical properties, also the sensitivity of the species can affect the ability to bioaccumulate pollutants. Different species have different potential to bioaccumulate compounds, even when they are exposed to the same levels of specific pollutants. Even individuals of one species exposed to the same concentration of contaminants in for similar period of time may not accumulate the substance at the same rate. It is associated with other factors such as age, sex, size, and physiological state of the organism (Garnaga 2012).

Information on the concentration levels of chemicals in treated effluents is insufficient to assess the risk to aquatic ecosystems. The results of chemical analysis of target and non-target compounds provide only some information about the potential hazard to humans and the environment. In addition, analysis of non-target compounds presents many difficulties for the analyst. Due to the fact that in treated sewage a complex mixture of compounds is present and also degradation and transformation products of these compounds are occurring, it is difficult to predict the effects of this type of bottom-up approach, based on criteria for individual chemicals (Fang et al. 2017). Many compounds present in treated effluents exhibit toxic properties. Therefore, the main detrimental effects of organic micropollutants are attributed to their potential acute toxicity or sub-lethal effects on the biota. Ecotoxicological studies seem to be an excellent tool for assessing the hazards arising from the presence of harmful compounds in the treated wastewater. The ecotest results reflect the actual threat to organisms occurring in certain ecosystems. What is more, they are less time consuming and do not require highly specialized analytical equipment and staff.

Ecotoxicity tests are performed on a biological sample, i.e., a representative population of a given species of organism, which is (or is not) a subject to some change/modification after exposure to the particular pollutant for a certain time. In ecotoxicological studies various bioassays, based on aquatic organisms, are used such as bacteria, algae, macrophytes, molluscs, crustacean, and fish. It is recommended to perform several tests incorporating various species that represent different trophic levels. The test result is based on the determination of the dose or concentration of the chemical substance inducing the specific effect on the indicator organism (e.g., LC_50_—lethal concentration which causes death in 50% individuals in the population, EC_50_—effect concentration which causes a measurable effect in 50% of individuals in population, IC_50_—inhibition concentration that causes growth suppression of 50% of individuals in population). In Table 2, toxicity of the compounds, which were identified at the highest concentration levels in the treated wastewater (see Table 1), towards the selected indicator organisms is given. This group of compounds is mainly dominated by pharmaceuticals.

**Table 2:** Toxicity parameters of the compounds which were identified at the highest concentration levels in the treated wastewater

Name of the compound	Parameter measured, duration of the test	Toxicity (mg/L)	Species	Additional information	References
Citalopram	EC_50_, 72 h	3.300	*Pseudokirchneriella subcapitata*		Minguez et al. (2016)
	EC_50_, 48 h	1.600	*Pseudokirchneriella subcapitata*	Growth inhibition	Cunha et al. (2019)
	EC_50_, 72 h	0.505	*Skeletonema marinoi*		Minguez et al. (2016)
	EC_50_	3.9	*Ceriodaphnia dubia*		Cunha et al. (2019)
	LOEC, 8 days	4.0	*Ceriodaphnia dubia*	Reproduction	
	EC_50_	30.14	*Daphnia similis*		
	EC_50_, 48 h	30.14	*Daphnia magna*		Minguez et al. (2016)
	EC_50_, 24 h	22.81	*Daphnia magna*		Yang et al. (2017)
	EC_50_, 48 h	7.44	*Daphnia magna*		
	EC_50_, 48 h	> 100	*Artemia salina*		Minguez et al. (2016)
	LOEC	0.000405	*Leptoxis carinata*		Cunha et al. (2019)
Gabapentin	LD_50_, 96 h	50	*Oncorhynchus mykiss*		Pfizer (2011)
	EC_50_, 24 h	> 500	*Daphnia magna*		
	EC_50_, 48 h	> 100	*Daphnia magna*		Minguez et al. (2016)
	EC_50_, 72 h	> 100	*Pseudokirchneriella subcapitata*		
	EC_50_, 48 h	> 100	*Artemia salina*		
	EC_50_, 72 h	>100	*Skeletonema marinoi*		
Tramadol	LC_50_, < 96 h	130	Unspecified fish	Tramadol HCl	Sanderson and Thomsen (2009)
	EC_50_, 48 h	73	*Daphnia spp.*	Tramadol HCl	
	EC_50_, 24 h	170	*Daphnia magna*		Le et al. (2011)
	EC_50_	> 4000	*Pseudomonas putida*	Tramadol HCl	Bergheim et al. (2012)
Lamivudine	EC_50_, 72 h	49.06	*Pseudokirchneriella subcapitata*	In pharmaceutical product, Kivexa with formoterol and abacavir	Guo et al. (2015)
	EC_50_, 72 h	> 96.9	*Pseudokirchneriella subcapitata*	In pharmaceutical product Zeffix	
	EC_50_, 72 h	96.9	*Pseudokirchneriella subcapitata*	In pharmaceutical product Epivir	
Efavirenz	EC_50_, 72 h	>0.012	*Pseudokirchneriella subcapitata*	In pharmaceutical product Stocrin	Guo et al. (2015)
	EC_50_	0.012–96.9	Green algae		
	EC_10_, 12 days	>0.76	*Microcystis aeruginosa*	In pharmaceutical product Stocrin	
	96 h	1.03 × 10^−5^	*Oreochromis mossambicus*	Liver damage, histology-based assessment	Robson et al. (2017)
Valsartan	NOEC, 72 h	85	*Desmodesmus subspicatus*		Perrodin and Orias (2017)
	LC_50_,96 h	> 100	*Salmo gairdneri* (*Oncorhynchus mykiss*)		Bayer et al. (2014)Oncorhynchus
	EC_50_, 48 h	> 580	*Daphnia magna*		
	EC_50_, 48 h	> 100	*Daphnia magna*		Minguez et al. (2016)
	EC_50_, 72 h	> 100	*Pseudokirchneriella subcapitata*		
	EC_50_, 48 h	> 100	*Artemia salina*		
*N*-acetyl-4-aminoantipyrine	LC_20_	10	*Daphnia magna*		Gómez et al. (2008)
4-Methyl-1H-benzotriazole	NOEC	> 15	*Oncorhynchus mykiss* (epithelial cell lines)	In mixture (1:1) with 5-methyl-1H-benzotriazole	Zeng et al. (2016)
	EC_50_, 15 min	21	*Vibrio fischeri*		Pillard et al. (2001)
	LC_50_, 48 h	118	*Ceriodaphnia dubia*		
	LC_50_, 96 h	63	*Pimephales promelas*		
Diclofenac	EC_50_	1950	*Raoultella* sp, strain DD4		Domaradzka et al. (2016)
	EC_50_, 15 min	14.31	*Vibrio fischeri*	Sodium salt	De García et al. (2014)
	EC_50_	416.67	*Staphylococcus warneri*		Domaradzka et al. (2016)
	MTC (microbial toxic concentration)	> 1300	*Pseudomonas aurantiaca* *Serratia rubidaea*		
	EC_50_	782.11	*Sorghum bicolor*	Sodium salt	Wieczerzak et al. (2018)
	EC_50_, 7 days	7.5	*Lemna minor*	Total frond area	Cleuvers (2003)
	EC_50_, 3 days	72	*Desmodesmus subspicatus*	Sodium salt	
	EC_50_, 48 h	68	*Daphnia magna*	Sodium salt,	
Acesulfame	NOEC, 48 h	> 1000	*Daphnia magna*		Stolte et al. (2013)
	NOEC, 7 days	> 1000	*Lemna minor*		
	NOEC, 24 h	> 1000	*Scenedesmus vacuolatus*		
1H-benzotriazole	EC_10_, 72 h	1.18	*Desmodesmus subspicatus*		Seeland et al. (2012)
	EC_50_, 72 h	231	*Scenedesmus subspicatus*	Growth	Cantwell et al. (2015)
	EC_50_, 72 h	102	*Scenedesmus subspicatus*	Biomass	
	EC_50_, 15 min	41.65	*Vibrio fischeri*	Luminescence	
	LC_50_, 48 h	107	*Daphnia magna*	Immobilization	Seeland et al. (2012)
	EC_50_, 21 days	25.9-76.9	*Daphnia magna*	Reproduction	Cantwell et al. (2015)
	LC_50_, 48 h	102	*Ceriodaphnia dubia*	Mortality	Pillard et al. (2001)
	EC_50_, 48 h	15.8	*Daphnia galeata*	Immobilization	Seeland et al. (2012)
	LC_50_, 96 h	65	*Pimephales promelas*		Pillard et al. (2001)
	LC_50_, 96 h	25	*Pimephales promelas*	Mortality, static	Cantwell et al. (2015)
	LC_50_, 48 h	25.7	*Pimephales promelas*	Mortality, static	
	LC_50_, 48 h	27.5	*Lepomis macrochirus*	Mortality, juvenile, static	Cantwell et al. (2015)
	LC_50_, 96 h	25	*Lepomis macrochirus*	Mortality, juvenile, static	
	EC10,7 days	3.94	*Lemna minor*		Seeland et al. (2012)
	EC_50_, 16 days	8.3	*Chlorella sorokiniana*	Growth	Gatidou et al. (2019)
Sucralose	NOEC, 21 days	1800	*Daphnia magna*		Huggett and Stoddard (2011)
	EC_50_/LC_50_, 28 days	> 93	*Mysid shrimp*		
	EC_50_/LC_50_	> 1800	*Daphnia magna*		Tollefsen et al. (2012)
	EC_50_/LC_50_, 96 h	> 1800	*Green algae*		
	NOEC, 7 days	114	*Lemna gibba*		
	NOEC, 28 days	93	*Americamysis bahia*		Huggett and Stoddard (2011)
Irbesartan	EC_50_, 96 h	460	*Pseudokirchneriella subcapitata*	In pharmaceutical product Aprovel	Guo et al. (2015)
	EC_50_, 48 h	> 100	*Daphnia magna*		Minguez et al. (2016)
	EC_50_, 72 h	> 100	*Pseudokirchneriella subcapitata*		
	EC_50_, 48 h	> 100	*Artemia salina*		
Iopromide	EC_50_, 72 h	10 000	*Pseudokirchneriella subcapitata*	In pharmaceutical product Ultravist	Guo et al. (2015)
	EC_50_, 48 h	> 1000	*Daphnia magna*		Santos et al. (2010)
	NOEC, 28 days	> 100	*Danio rerio*		
Darunavir	EC_50_	43-100	Green algae		Guo et al. (2015)
	EC_50_, 72 h	> 43	*Pseudokirchneriella subcapitata*	In pharmaceutical product Prezista	
Bromazepam	LOEC	1.5	*Danio rerio*		Gebauer et al. (2011)
	LOEC/21-23 days	0.0001	*Daphnia magna*		Rivetti et al. (2016)
	LOEC 48 h	0.0001	*Daphnia magna*		
Naproxen	EC_50_, 48 h	174	*Daphnia magna*	Naproxen-sodium	Cleuvers (2003)
	EC_50_, 48 h	43.64	*Ceriodaphnia dubia*	Naproxen-sodium	Isidori et al. (2005)
	EC_50_, 48 h	66.37	*Ceriodaphnia dubia*		
	LC_50_, 24 h	43.54	*Thamnocephalus platyurus*	Naproxen-sodium	
	LC_50_, 24 h	84.09	*Thamnocephalus platyurus*		
	EC_50_, 96 h	2.68	*Hydra attenuata*		Quinn et al. (2008)
	LC_50_, 24 h	54.64	*Brachionus calyciflorus*	Naproxen-sodium	Isidori et al. (2005)
	EC_50_, 7 days	24.2	*Lemna minor*	Naproxen-sodium	Cleuvers (2003)
	EC_50_, 96 h	3.7	*Pseudokirchneriella subcapitata*		Harada et al. (2008)
	IC_50_, 96 h	39.31	*Pseudokirchneriella subcapitata*	Naproxen-sodium	Isidori et al. (2005)
	EC_50_, 3 days	> 320	*Desmodesmus subspicatus*	Naproxen-sodium	Cleuvers (2003)
	EC_50_, 15 min	18.5	*Vibrio fischeri*		Harada et al. (2008)
	EC_50_, 15 min	17.92	*Vibrio fischeri*		De García et al. (2014)
Acetaminophen	EC_50_, 48 h	> 160	*Oryzias latipes*		Kim et al. (2007)
	EC_50_, 96 h	> 160	*Oryzias latipes*		
	LC_50_, 96 h	26.6	*Daphnia magna*		
	LC_50_, 48 h	30.1	*Daphnia magna*		
	EC_50_, 48 h	34.99	*Daphnia magna*		Minguez et al. (2016)
	EC_50_, 5 min	549.7	*Vibrio fischeri*		Kim et al. (2007)
	EC_50_, 15 min	657.5	*Vibrio fischeri*		
	EC_50_, 15 min	363.30	*Vibrio fischeri*		De García et al. (2014)
	EC_50_, 72 h	> 100	*Pseudokirchneriella subcapitata*		Minguez et al. (2016)
	EC_50_, 48 h	> 100	*Artemia salina*		
Diatrizoate	NOEC	613.92–0.61392 × 10^−5^ (10^−3^–10^−11^ M)	*Tetrahymena pyriformis*	Na-diatrizoate	Láng and Kőhidai (2012)
	NOEC	613.92 (0.001 M)	Ciliate	Population growth	Perrodin and Orias (2017)
Caffeine	EC_50_	290.2	*Desmodesmus subspicatus*	Population growth	Kobetičová et al. (2015)
	IC_50_	265	*Sinapis alba*	Root length	
Metronidazole-OH	IC_50_, 30 min	> 100	*aerobic bacteria*	Metronidazole	Kümmerer et al. (2004)
	IC_50_, 20 h	> 100	*aerobic bacteria*	Metronidazole	
	EC_50_	243	*Vibrio fischeri*	Metronidazole	Kołodziejska et al. (2013)
	EC_50_	> 64 000	*Pseudomonas putida*	Metronidazole	Kümmerer et al. (2004)
Metformin	EC_50_, 48 h	64	*Daphnia magna*		Cleuvers (2003)
	EC_50_, 3 days	> 320	*Desmodesmus subspicatus*		
	EC_50_, 7 days	110	*Lemna minor*		

The highest toxicity to *Pseudokirchneriella subcapitata* microalgae was noted for citalopram and naproxen, while for gabapentin, valsartan, irbesartan, and acetaminophen it was the lowest. High sensitivity to naproxen and its compounds also show *Vibrio fischeri* bacteria, *Hydra attenuate,* and *Lemna minor.* A source of high toxicity to *Lemna minor* was also diclofenac and benzotriazole. However, acesulfame is not toxic to *Lemna minor*.

Acetaminophen exhibits high toxicity to *Daphnia magna*, whereas its toxicity to the bacteria *Vibrio fischeri* is low. In turn, gabapentin manifests low toxicity with respect to all the examined indicator organisms. *Vibrio fischeri* bacteria are also sensitive for diclofenac but responsive to metronidazole (see Table 2). Studies reported that X-ray contrast media (i.e., iopromide, iopamidol) did not pose risk to aquatic organisms at environmentally relevant concentrations; however, data on combined toxic effects between X-ray contrast media and other substances present in environment are still scarce (Haiß and Kümmerer 2006; Tran et al. 2018).

Many of the compounds identified in wastewater have the potential to disrupt endocrine processes. Endocrine-disrupting chemicals (EDCs) are substances naturally or anthropogenically occurring in the environment. According to the definition, adopted by World Health Organization (WHO), they are exogenous compounds or mixtures with properties to change the function of the endocrine system, which will result in negative consequences on the organism, its progeny, or subpopulations. These compounds belong to different chemical families, and are able to interfere with the hormonal system of exposed organisms by mimicking or counteracting natural hormones (Huerta et al. 2016). It has been estimated that from hundreds of thousands of presently produced compounds around 1000 of them may have endocrine-disrupting properties (Gore et al. 2014). Those compounds include mainly polychlorinated biphenyls (PCBs), bisphenol A, phthalates, pesticides, some pharmaceuticals, brominated flame retardants, and organic tin compounds (Kima et al. 2015). The standard method for biological treatment of wastewater used in a typical wastewater treatment results in only partial removal of the compounds from the group of EDCs, mainly of polar nature (Välitalo et al. 2016). EDCs are detected both in surface and ground waters. This phenomenon is alarming due to the fact that those compounds, when released into the water, may adversely affect living organisms, even if they occur at low levels (Kima et al. 2015). There are many literature reports which indicate that EDCs can cause adverse effects on the aquatic environment even at low concentrations. For example, studies have shown that Zebrafish were sensitive to estradiol at a very low concentration of 0.2 ng/L (Westerlund et al. 2000). The EDCs compounds identified in treated effluents include phthalate compounds such as bis (2-ethylhexyl) benzene-1,2-dicarboxylate (DEHP) and benzyl butyl benzene-1,2-dicarboxylate (BBP) and phenols such as 4-*tert*-octylphenol and bisphenol A (BPA). These compounds are included at the European Commission priority lists of 66 endocrine active substances for which clear evidence of endocrine-disrupting activity is confirmed (Category I) (EC 2016).

An alternative for classical analytical methods for the determination of endocrine active substances are endocrine tests. These bioassays can thus be used to determine total estrogenic activities in (extracts of) environmental samples, without the necessity of knowing all compounds present that contribute to the activity (Houtman et al. 2007). The most commonly used tests include reporter gene assays such as the yeast estrogen/androgen screen (YES/YAS), which allows identification of both, the activation (agonist) or inhibiting (antagonist) properties in samples of wastewater or estrogen receptor-mediated chemical-activated luciferase gene expression (ER-CALUX^®^) assay (Houtman et al. 2007).

Exposure to chemical substances may cause damage of the genetic material of the organisms. Genotoxic compounds acting directly or indirectly on the body have the potential for altering the organism’s genetic code. In addition, such compounds can induce changes not only within one generation. Effects of their action can be observed over an extended period of time, across the whole population. Therefore, it is important to carry out genotoxicity studies, especially in the case if a particular ecosystem is exposed to the constant supply of pollutants.

EDCs in wastewater effluents may be leached from microplastics (Anderson et al. 2016). Microplastics pollution is a high and increasing concern in European Union (SAM 2018). The term microplastics refers to the group of organic polymers derived from various petroleum compounds with the upper size limit of 5 mm. Studies indicated that wastewater treatment plants (WWTPs) play an important role in releasing microplastics to the environment. The growing concerns about microplastics presence in wastewater effluents and subsequently in marine environment have been attributed to their ubiquity, long residence times accompanied by difficult removing, possibility of being assimilated by living organisms, and thus entering trophic levels as well as easiness to undergo numerous transformations during wastewater treatment process (Anderson et al. 2016; Sun et al. 2019). WWTP are not designed to fully remove microplastics, and its removal depends on the treatment process applied; however, in most cases it exceeds 80–90%. An average microplastics concentrations reviewed in literature is in the range of 0–447 particles/L (Sun et al. 2019).

## Conclusion

Over the last years, the issue of water quality has gained strategic importance, both in the European Union and internationally. The challenge of the present day is to protect effectively aquatic ecosystems, to preserve their good condition and to reduce negative impacts on human health. The purpose of wastewater treatment plant is to remove compounds that may have adverse effects on the environment and on human health, but as the research shows the processes used in wastewater treatment are insufficient. As a result, compounds potentially hazardous may enter the surface waters. Legislation aimed at eliminating/reducing emissions to the environment are restricted to a narrow spectrum of chemicals. Most of the compounds remain beyond the legal norms.

As we have shown in our paper, the interest in the presence of micropollutants in wastewater has been reflected in the research carried out. Studies on the quality evaluation of treated effluents are carried out in a number of research centers around the world. They mainly focus on the determination of the target compounds and relate to pharmaceuticals such as analgesic/anti-inflammatories or antibiotics. Carried out literature review on the highest concentrations of contaminants in treated effluents indicates that they are observed for analgesic/anti-inflammatory drugs, i.e., diclofenac, tramadol naproxen, antiretroviral agents, industrial chemicals, or contrast media. These compounds occur at levels of several tens of μg/L. Although the number of studies on the determination of target pollutants in the treated wastewater is constantly increasing, there are few reports in literature on the identification of non-target compounds present in the treated wastewater. This is primarily due to the fact that such research is time consuming, requires a variety of analytical techniques, often complex or costly and sophisticated equipment. The classical methodology for assessing the environmental impact of xenobiotics, which is required by legislation, is based on the use of chemical analysis techniques, which allow to determine the concentrations of pollutants in environmental samples. However, the thus obtained results do not adequately reflect the risks to ecosystems and subsequently humans. On the basis of chemical analysis, the possible interactions between toxic substances and their mixture effects on living organisms cannot be determined. Therefore, the methodology for assessing the quality of treated wastewater should include, in addition to chemical analyses, ecotoxicological, genotoxic/mutagenic, or endocrine activity studies. In this way, comprehensive information on the hazard arising from the presence of all known and unknown hazardous substances in the treated wastewater is needed. Moreover, it should be noted that during wastewater treatment various by-products, of unknown properties and toxicity may be formed. Therefore, when developing new, more effective methods of wastewater treatment, it is necessary to evaluate the toxicity of the resulting products. What is more, the indicator organisms selected for ecotoxicity evaluation should be from different trophic levels as various organisms may exhibit a diverse sensitivity to the compounds, e.g., naproxen is highly toxic to *Hydra attenuata* and its toxicity to *Daphnia magna* is low. Researchers from the Institute for Inland Water Management and Water Treatment (RIZA) have already mentioned the need for a comprehensive assessment of waste water quality in the 1990s. A method for whole-effluent assessment contained a series of tests to make (potential) effects visible, focusing on the following five parameters: acute and chronic toxicity, bioaccumulation, mutagenicity, and persistence.

The problem of occurrence of potentially hazardous compounds in wastewater and surface waters to which wastewater is being discharged has been observed in many countries. The first country in which legal regulations were issued, mandating the implementation of the subsequent stage of sewage treatment intended to remove the micropollutants is Switzerland. Similar actions have been taken in Germany. Therefore, it seems that the introduction of wastewater treatment technology of micropollutants removal in other European countries is only a matter of time.
